# Single-Molecule Dynamics at a Bacterial Replication Fork after Nutritional Downshift or Chemically Induced Block in Replication

**DOI:** 10.1128/mSphere.00948-20

**Published:** 2021-01-27

**Authors:** Rogelio Hernández-Tamayo, Hannah Schmitz, Peter L. Graumann

**Affiliations:** a SYNMIKRO, LOEWE Center for Synthetic Microbiology, Marburg, Germany; b Department of Chemistry, Philipps Universität Marburg, Marburg, Germany; University of Wyoming

**Keywords:** replication, single-molecule microscopy, DNA primase, helicase, stringent response, *Bacillus subtili*s, DNA helicase, DNA polymerase, DNA replication, single-molecule tracking

## Abstract

Replication forks must respond to changes in nutrient conditions, especially in bacterial cells. By investigating the single-molecule dynamics of replicative helicase DnaC, DNA primase DnaG, and lagging-strand polymerase DnaE in the model bacterium Bacillus subtilis, we show that proteins react differently to stress conditions in response to transient replication blocks due to DNA damage, to inhibition of the replicative polymerase, or to downshift of serine availability. DnaG appears to be recruited to the forks by a diffusion and capture mechanism, becomes more statically associated after the arrest of polymerase, but binds less frequently after fork blocks due to DNA damage or to nutritional downshift. These results indicate that binding of the alarmone (p)ppGpp due to stringent response prevents DnaG from binding to forks rather than blocking bound primase. Dissimilar behavior of DnaG and DnaE suggests that both proteins are recruited independently to the forks rather than jointly. Turnover of all three proteins was increased during replication block after nutritional downshift, different from the situation due to DNA damage or polymerase inhibition, showing high plasticity of forks in response to different stress conditions. Forks persisted during all stress conditions, apparently ensuring rapid return to replication extension.

**IMPORTANCE** All cells need to adjust DNA replication, which is achieved by a well-orchestrated multiprotein complex, in response to changes in physiological and environmental conditions. For replication forks, it is extremely challenging to meet with conditions where amino acids are rapidly depleted from cells, called the stringent response, to deal with the inhibition of one of the centrally involved proteins or with DNA modifications that arrest the progression of forks. By tracking helicase (DnaC), primase (DnaG), and polymerase (DnaE), central proteins of Bacillus subtilis replication forks, at a single molecule level in real time, we found that interactions of the three proteins with replication forks change in different manners under different stress conditions, revealing an intriguing plasticity of replication forks in dealing with replication obstacles. We have devised a new tool to determine rates of exchange between static movement (binding to a much larger complex) and free diffusion, showing that during stringent response, all proteins have highly increased exchange rates, slowing down overall replication, while inactivation of polymerase or replication roadblocks leaves forks largely intact, allowing rapid restart once obstacles are removed.

## Abstract (precis)

All cells need to adjust DNA replication, which is achieved by a well-orchestrated multiprotein complex, in response to changes in physiological and environmental conditions. For replication forks, it is extremely challenging to meet with conditions where amino acids are rapidly depleted from cells, called the stringent response, to deal with the inhibition of one of the centrally involved proteins or with DNA modifications that arrest the progression of forks.

## INTRODUCTION

DNA replication is a highly choreographed and tightly regulated event in the life cycle of all cells. It is carried out by a dynamic, multiprotein complex known as the replisome, which precisely coordinates the action of several distinct factors to efficiently and rapidly couple DNA unwinding with high-fidelity nucleic acid synthesis (1, 2). Importantly, DNA replication must respond to situations of changing environmental or developmental conditions, including response to damage in the template and the reduced nutritional capacity of cells (3). This is especially relevant for bacterial cells that are directly affected by changes in their surroundings.

DNA replication is inherently asymmetric; one daughter strand, termed the leading strand, is continually synthesized in the same direction as the unwinding of the DNA duplex. The other (lagging) strand is synthesized in the opposite direction in short intervals, giving rise to Okazaki fragments (4). In contrast to the model bacterium Escherichia coli and many other species, which use the same polymerase at both strands (5, 6), the Gram-positive model organism Bacillus subtilis and *Firmicutes* in general use two essential DNA polymerases, PolC and DnaE, for replication (7, 8). *In vivo*, PolC is the main replicative polymerase, while DnaE acts in lagging-strand synthesis. *In vitro* reconstitution of the B. subtilis replisome has demonstrated that PolC is responsible for all leading-strand synthesis as well as most lagging-strand synthesis, whereas the more error-prone and much slower DNA replicase DnaE (25 to 60 nucleotides [nt]/s for DnaE compared to ∼500 nt/s for PolC) plays a crucial role in initiating lagging-strand synthesis (9, 10). DnaE is important for extending the lagging-strand RNA primer before handing off to PolC, which then completes replication of the Okazaki fragment (10–12). The synergistic relationship between two polymerases in the B. subtilis replisome resembles the synergy found in eukaryotic replication (13, 14).

Replication of the lagging strand requires a specialized RNA polymerase, termed primase, to initiate each Okazaki fragment with a short oligoribonucleotide (15). In B. subtilis the primase, DnaG, is recruited to the replication fork by an interaction with the replicative helicase, DnaC, where it synthesizes an RNA primer every 1.5 to 2 kb (10). Working together, the helicase and primase unwind the DNA template and initiate thousands of regularly spaced Okazaki fragments to promote fork progression at a rate of 1,000 bases per second in rapidly dividing bacterial cells. The direct association of primase and helicase coregulates their functions. For example, primase increases both the ATPase and helicase activities of DnaC. Similarly, DnaC can modulate the overall activity of DnaG as well as the length of primers synthesized by primase and its initiation specificity (12). Each Okazaki fragment is initiated by a short RNA primer by DnaG and likely is extended for a few base pairs by DnaE, which then hands over to PolC (16). Primase requires interaction with the helicase to stimulate its RNA polymerase activity (17). In E. coli, the helicase-primase contact is established through the interaction between the C-terminal domain of primase (18, 19) and the N-terminal domain of the helicase (20). In E. coli, this interaction is weak and transient, with a dissociation constant in the low micromolar range, giving rise to fast on/off kinetics (19). However, in Geobacillus stearothermophilus, helicase and primase form a stable complex that can be isolated and crystallized (21).

Bacteria respond to nutrient downshift by the so-called stringent response. Noncharged tRNAs arise when a shortage of any amino acid occurs and bind to the A-site on the ribosome, where they are sensed by RelA (22, 23). This multifunctional enzyme in turn is activated and converts GTP into ppGpp (guanosine 3′,5′-bispyrophosphate) or pppGpp (guanosine 3′-diphosphate 5′-triphosphate), a second messenger that triggers many events, including downregulation of translation, and increasing the synthesis of enzymes for, e.g., amino acid synthesis pathways (24). Interestingly, (p)ppGpp also binds to DnaG, whereby DNA replication is greatly slowed, or even stopped, until nutrient shortage is overcome (25, 26). The stringent response induces arrest of DNA replication in B. subtilis and, to some extent, in E. coli (27, 28). The interference of (p)ppGpp with the activity of DNA primase inhibits replication elongation in a dose-dependent manner and adjusts the elongation rate according to the nutritional status of the cell (25, 26).

It has been unknown if replication forks dissipate or adapt during the stringent response, so we have captured changes in single-molecule dynamics of functional fluorescent protein fusions generated for DnaC, DnaG, and DnaE after inhibition of serine tRNA synthetase. We also monitored exchange rates of the three proteins acting on the lagging strand occurring in response to DNA damage-induced blocks of replication forks or by specifically blocking PolC. We observed distinct changes at the single-molecule level to the three scenarios described above, showing that replication forks have a high plasticity to deal with different stress situations in a bacterial cell.

## RESULTS

We wished to gain insight into the changes of dynamics occurring at Bacillus subtilis replication forks in response to conditions inducing a transient block in DNA replication, including the response to nutritional downshift, termed stringent response. Our strategy was to employ serine hydroxamate (SHX) to induce the stringent response (SHX blocks serine tRNA synthetase, leading to an accumulation of uncharged serine tRNAs) (29) and to compare dynamics of replication proteins to those seen after addition of mitomycin C (MMC), which induces DNA damage supposed to transiently block the progression of forks (13, 30), or of 6(p-hydroxyphenylazo)-uracil (HPUra), which reversibly binds to and inhibits DNA polymerase PolC, thereby blocking progression of replication (31).

We additionally generated functional mVenus (mV) fluorescent protein fusions to DnaC and to DnaG and employed fusions to DnaE and to DnaX, previously shown to be able to functionally replace wild-type proteins (13). In contrast to the other fusions, DnaX-cyan fluorescent protein (CFP) was expressed from the ectopic *amyE* site, generating a merodiploid strain for this fusion. We found that a fusion of mVenus to the C terminus of DnaC or to that of DnaG, each expressed from the original gene locus under native transcriptional control, as the sole source of the protein in the cell (Fig. S1E) did not negatively affect exponential growth of B. subtilis cells (Fig. S1A, B, and C). Additionally, treatment of cells expressing each fusion with MMC, HPUra, or SHX showed survival like cells lacking any of the protein fusions (Fig. S1H). Note that DnaE-mVenus-expressing cells showed a weak sensitivity toward SHX or MMC treatment (Fig. S1H). Additionally, we analyzed the chromosome content of exponentially growing cells by flow cytometry. Cells were stained with Vybrant DyeCycle orange, and 20,000 cells were analyzed per strain. Figure S1G shows that all cells showed variations between one, two, and more chromosome copy contents but overall had comparable patterns. Note that the number of chromosome copies per cell varies greatly within an exponentially growing B. subtilis culture (32), likely explaining differences between the analyzed strains. Taken together, we note that all fusions appear to largely complement the function of wild-type proteins.

10.1128/mSphere.00948-20.1 FIG S1 (A to D) Survival assay. Cultures of BG214, DnaC-mVenus, DnaE-mVenus, and DnaG-mVenus strains, before and after treatment with MMC, HPUra, or SHX, with concentrations as indicated in the corresponding legend. All strains were grown in duplicate in three independent experiments at 30°C. (E) Western blot of fluorescent protein fusions. Shown are Western blots from whole-cell lysates of (1) E. coli expressing mVenus, (2) B. subtilis BG214, or (3) DnaC-mVenus-, (4) DnaE-mVenus-, and (5) DnaG-mVenus-expressing cells. Corresponding protein sizes are indicated by arrowheads. Proteins were probed using a 1:500 dilution (rabbit-α-GFP), and secondary antibody was added (goat-α-rabbit-antibody in 1:10,000 dilution) after a series of washing steps. Cells were harvested in exponential phase at an OD_600_ of 0.5 to 0.7 prior to analysis. (F) Western blot analysis after drug addition revealing no differences in protein levels under any experimental condition. Cultures of DnaC-mVenus, DnaE-mVenus, DnaG-mVenus, and YFP-MreB strains, before and after treatment with MMC, 50 ng/ml, 6(p-hydroxyphenylazo)-uracil (HPUra), 50 μg/ml, or d,l-serine hydroxamate (SHX), 5 mg/ml. Cells were harvested in exponential phase at an OD_600_ of 0.5 to 0.7 prior to analysis. Proteins were probed using a 1:500 dilution (rabbit-α-GFP), and secondary antibody was added (goat-α-rabbit-antibody in 1:10,000 dilution) after a series of washing steps. (G) Flow cytometry analysis for DNA content in cells of indicated genotypes. Cells were stained with Vybrant DyeCycle orange, and 20,000 cells were analyzed per strain. *y* axis, number of cells; *x* axis, fluorescence in arbitrary units (AU). Numbers on the *x* axis indicate chromosome numbers. (H) Spot assays. Cultures of BG214 or of strains expressing DnaC-mVenus, DnaE-mVenus, or DnaG-mVenus before and after treatment with MMC, 50 ng/ml, HPUra, 50 μg/ml, or SHX, 5 mg/ml. Download FIG S1, TIF file, 2.0 MB. Copyright © 2021 Hernández-Tamayo et al. 2021 Hernández-Tamayo et al. This content is distributed under the terms of the Creative Commons Attribution 4.0 International license.

**FIG 1:**
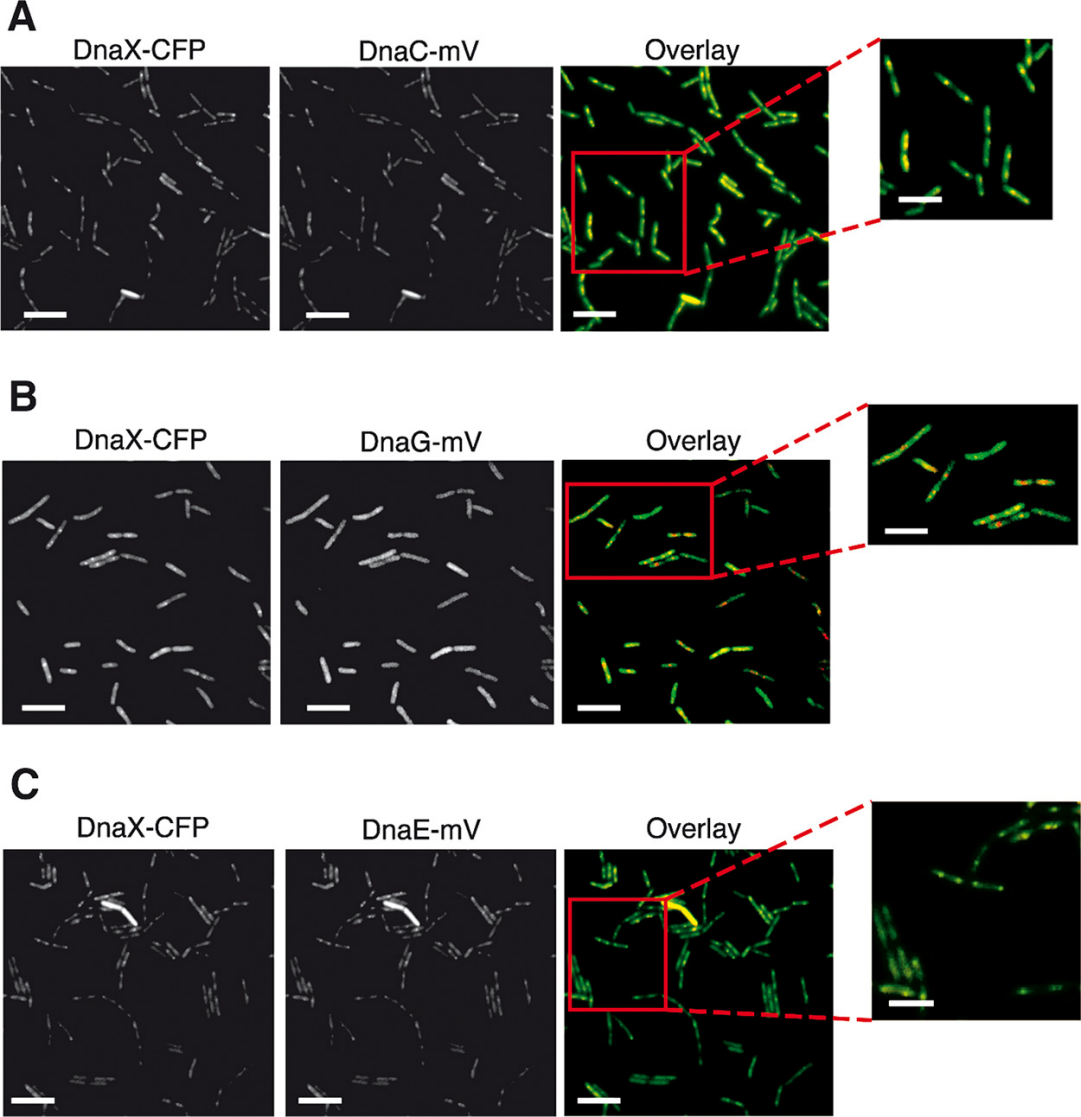
Colocalization of DnaC-mVenus, DnaE-mVenus, or DnaG-mVenus with DnaX-CFP in B. subtilis cells. Epifluorescence microscopy of cells expressing both DnaC-mV, or DnaE-mV, or DnaG-mV, and DnaX-CFP during exponential growth as sole sources of the proteins. Scale bars, 5 μm; zoom panel, 2 μm.

Using high-resolution epifluorescence microscopy, we found that, very similar to DnaX-CFP, DnaC-mVenus formed fluorescent foci within the cells (Fig. 1A). In 80% of cases, DnaX-CFP and DnaC-mVenus foci colocalized (in the rest of events, only one of the two signals were visible), showing that the helicase fusion is recruited to replication forks, as expected.

For DnaG, two principle scenarios could be envisioned: (i) DnaG may come and go to forks by a diffusion/capture mechanism, which would lead to an exchange event every few thousand base pairs, and (ii) DnaG might have binding sites at the forks (analogous to DnaA, the initiator of replication [33]), which would lead to an enrichment at the forks and a concomitant enhanced replacement efficiency through a local pool of molecules. Figure 1B shows that in most cells, DnaG-mVenus was dispersed throughout the cells, with some cells showing weak accumulations. Thus, at first sight, there does not seem to be an accumulation of DnaG at the forks.

**FIG 2:**
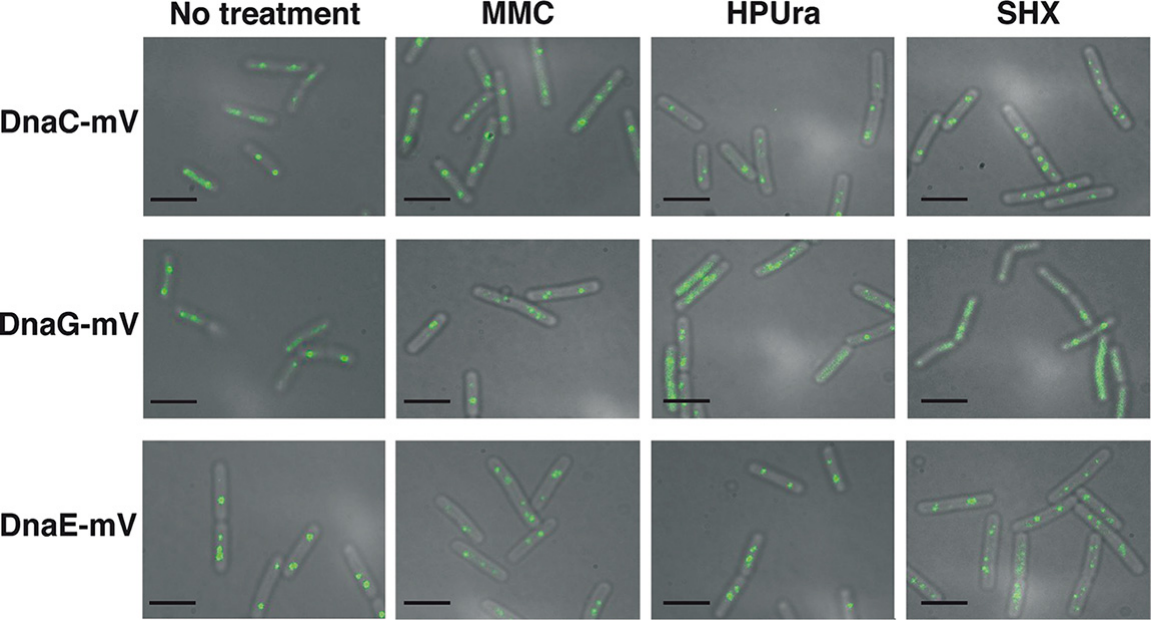
Localization of DnaC-mVenus, DnaG-mVenus, and DnaE-mVenus during stress conditions. Epifluorescence microscopy of B. subtilis cells expressing DnaC-mV, DnaG-mV, or DnaE-mV as the sole source of the proteins treated with different drugs as indicated above the panel rows. Scale bars, 2 μm.

Next, we treated cells with concentrations of MMC, HPUra, or SHX that led to a slowed growth rate but did not stop growth (Fig. S1A to C). We reasoned that these concentrations were able to strongly act at the respective targets, including replication forks, but did not lead to a large degree of cell death. Figure 2 shows that DnaC-mVenus foci appeared to be visually weaker after treatment with HPUra and SHX than after MMC treatment. As we will move on to single-molecule tracking (SMT) below, we will refrain from quantifying these results. For DnaG-mVenus, we noted the appearance of visible foci after addition of MMC and HPUra but not in response to SHX (Fig. 2). While we observed the described changes in localization patterns, the continued presence of foci representing replication forks for the three analyzed conditions is well apparent, indicating that, in general, forks persist through the three types of treatments.

### Single-molecule tracking reveals poor enrichment of DnaG at replication forks, arguing for a diffusion capture mechanism for recruitment.

**FIG 3:**
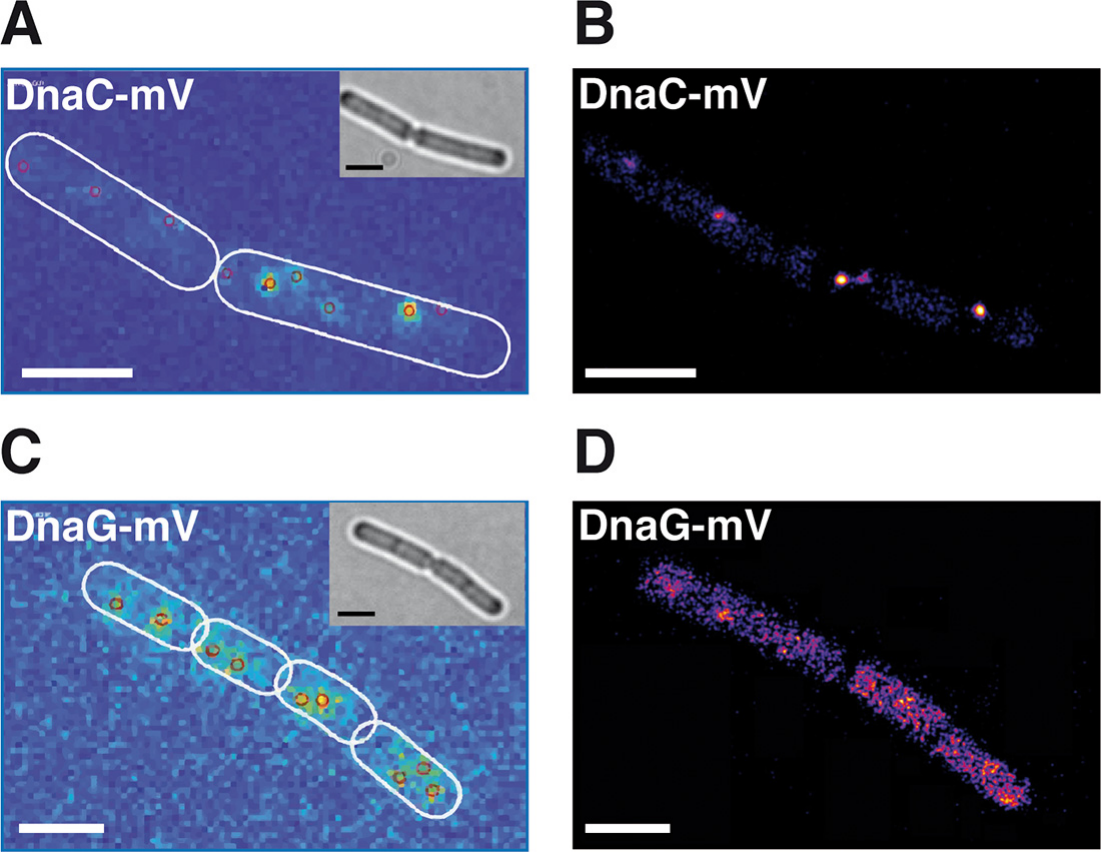
Sum of single-molecule tracking movies of exponentially growing cells expressing DnaC-mVenus or DnaG-mVenus. Insets in panels A and C show bright-field images, and outlines of cells are indicated by white ovals. Panels A and C show heat maps of localization, and panels B and D show fluorescence images. Please note that background was reduced in images from panels B and D using the “Image Background” tool in the Fiji program plugin “GDSC SMLS.” White bars, 2 μm.

**FIG 4:**
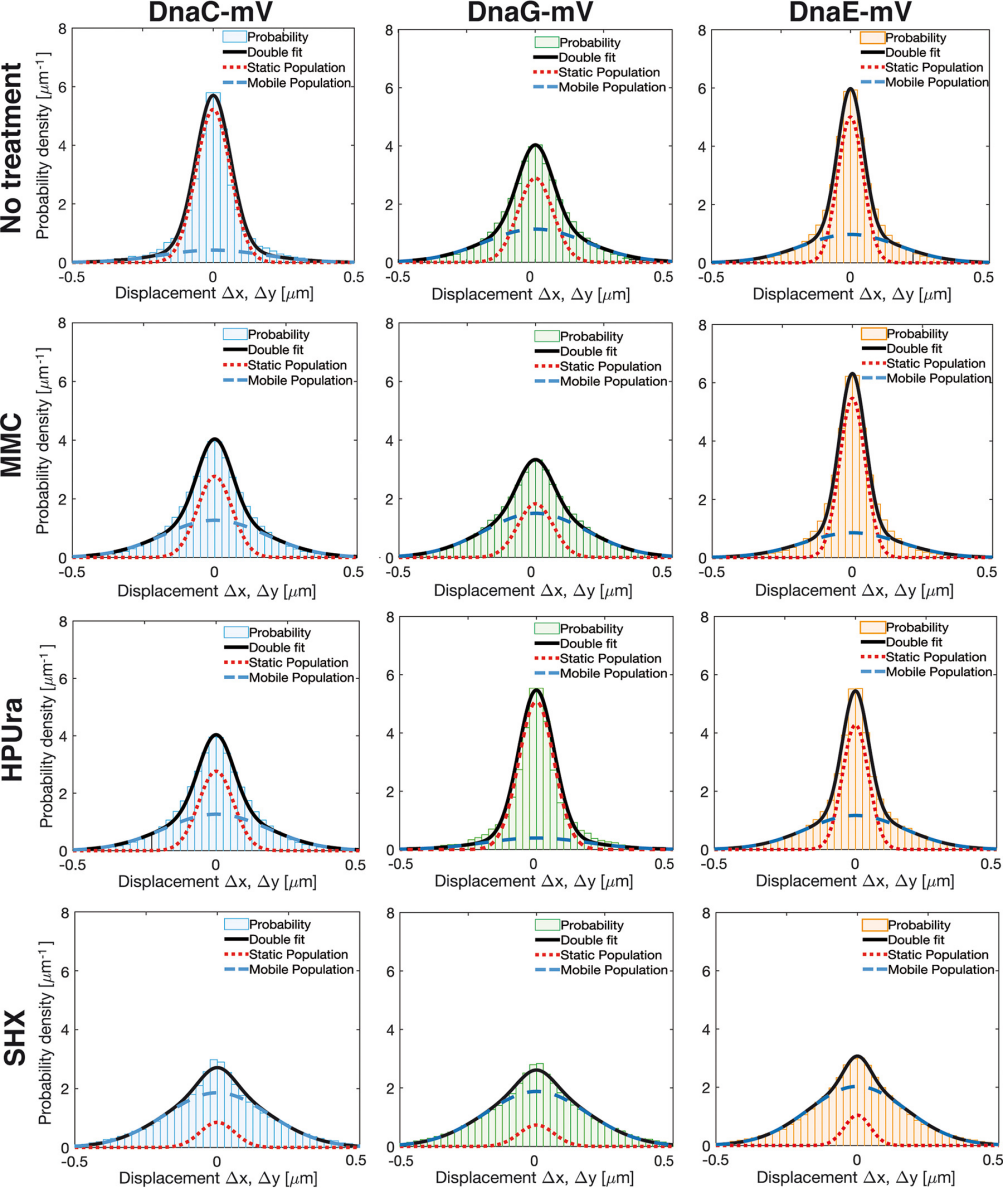
Diffusion patterns of DnaE-mVenus, DnaC-mVenus, and DnaG-mVenus under different conditions. Gaussian mixture model (GMM) analyses of frame-to-frame displacements in *x* and *y* directions. Black lines represent the sum of the two Gaussian distributions. Dotted red and blue lines represent the single Gaussian distributions corresponding to the static and mobile fractions.

**FIG 5:**
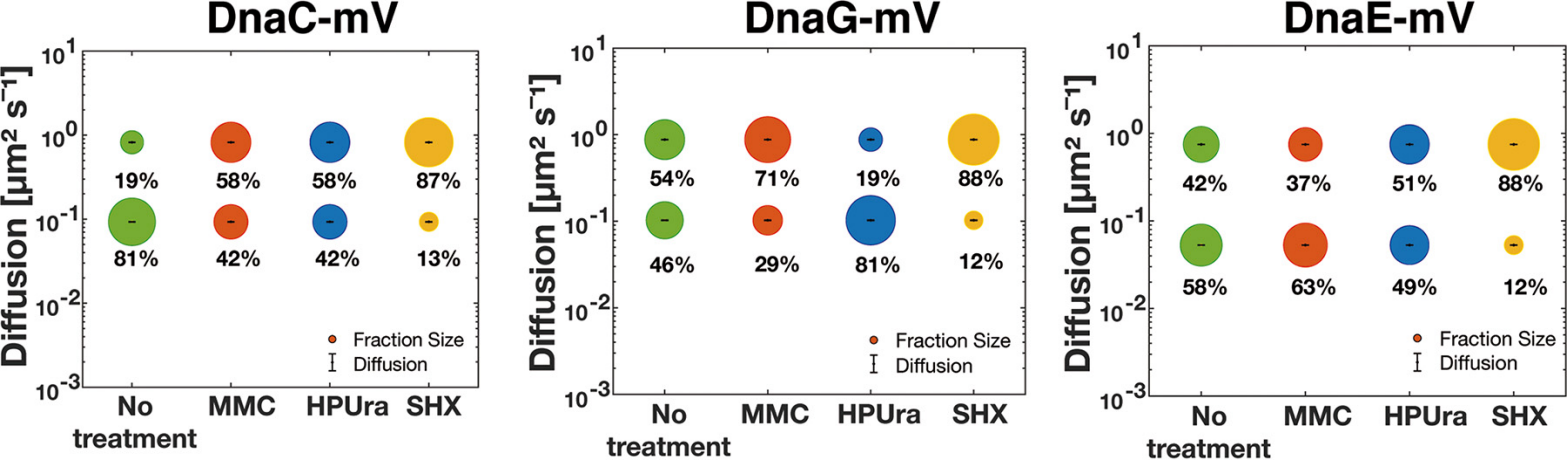
Diffusion patterns of DnaC-mVenus, DnaG-mVenus, and DnaE-mVenus. Gaussian mixture model (GMM) analyses of frame-to-frame displacements in *x* and *y* directions. Bubble plots show a comparison of fraction sizes (size of the bubble) and diffusion constants (*y* axis) between different growth conditions: distribution in untreated cells (green circles) and in MMC-treated (red circles), HPUra-treated (blue circles), and SHX-treated (yellow circles) cells. Step size distributions reveal two populations for each protein, a mobile (upper circles) and a static (lower circles) fraction.

We next turned to SMT to quantify changes in protein dynamics at the single-molecule level, using an experimental setup that has been described before (13). We used SMTracker software 1.5 (34) to analyze tracks that were generated using u-track software (35). We employed 20-ms stream acquisition to ensure that even freely diffusing molecules of DnaC (50.4 kDa plus mVenus) and of DnaG (68.6 kDa plus mVenus) would be trackable. Figure 3A and B show overlays of all frames of a typical movie (see Movies S1 and S2 for examples) of DnaC, revealing clear foci within cells that contain little background, indicative of a large number of DnaC molecules being present at the forks. Conversely, we observed barely detectable discrete accumulations of DnaG, but many molecules localized throughout the cells (Fig. 3C and D). Thus, these two proteins show clearly distinct localization patterns, indicating that DnaG only transiently and briefly associates with the replication machinery, contrary to DnaC. For quantification of molecule trajectories, we employed Gaussian mixture modeling (GMM), which allows us to directly compare molecule dynamics between different proteins or for a given protein between different growth conditions: a common value for the diffusion constant *D* is found, which leaves alterations only to occur in changes of the population size of molecules with a given diffusion coefficient. GMM also allows us to distinguish if the probability density function of observed step sizes can be explained by a single Gaussian function and, thus, by the presence of a single population of molecules having the same value for *D* or by two or three different populations, which is tested by an *r*^2^ analysis (13). Figure S2 shows that for all three proteins, observed distributions could be well explained by the existence of two populations but not of one. One fraction had a low diffusion constant, corresponding to molecules bound to replication forks, and one with a high value for *D*, characteristic of freely diffusing molecules, as was described before (13). Figure 4, upper row, illustrates that DnaC featured the highest proportion of statically bound molecules, with 81% (SD, 1.7%) (Fig. 5), and only 19% ± 1.3% diffusive molecules (Table 1), as was expected for the helicase. DnaE showed a relatively balanced proportion of 58% ± 2.2% for bound and 42% ± 1.6% for diffusive molecules (Fig. 5), while DnaG had the lowest population of static molecules (46% ± 1.9%) and a larger amount (54% ± 1.6%) of unbound ones. These data strongly suggest that DnaG comes and goes to forks and is only present at very low, likely single-molecule levels at the replication machinery, suggesting a diffusion capture model for recruitment to the lagging strands rather than an exchange with molecules prebound to other replication-associated proteins.

**TABLE 1:** Diffusion constants and percentages of static and mobile molecule fractions

Strain	No. of cells	No. of tracks	*D*^*a*^	*D*_1_^*b*^	*F*_1_^*c*^	*D*_2_^*d*^	*F*_2_^*e*^
Nontreated							
DnaE-mV	136	6,058	0.032 ± 0.004	0.053 ± 0.005	58 ± 2.2	0.75 ± 0.014	42 ± 1.6
DnaC-mV	122	5,303	0.039 ± 0.005	0.093 ± 0.004	81 ± 1.7	0.82 ± 0.025	19 ± 1.3
DnaG-mV	150	5,893	0.153 ± 0.007	0.100 ± 0.005	46 ± 1.9	0.88 ± 0.020	54 ± 1.6
MMC treated							
DnaE-mV	136	5,275	0.027 ± 0.005	0.053 ± 0.005	63 ± 1.7	0.75 ± 0.014	37 ± 2.2
DnaC-mV	134	5,139	0.107 ± 0.005	0.093 ± 0.004	42 ± 1.6	0.82 ± 0.025	58 ± 1.8
DnaG-mV	135	7,097	0.174 ± 0.007	0.100 ± 0.005	29 ± 1.4	0.88 ± 0.020	71 ± 1.5
HPUra treated							
DnaE-mV	121	4,775	0.093 ± 0.007	0.053 ± 0.005	49 ± 2.3	0.75 ± 0.014	51 ± 1.2
DnaC-mV	134	5,135	0.078 ± 0.005	0.093 ± 0.004	42 ± 1.5	0.82 ± 0.025	58 ± 1.5
DnaG-mV	110	5,526	0.109 ± 0.007	0.100 ± 0.005	81 ± 1.6	0.88 ± 0.020	19 ± 1.1
SHX treated							
DnaE-mV	158	5,067	0.220 ± 0.007	0.053 ± 0.005	12 ± 2.4	0.75 ± 0.014	88 ± 1.9
DnaC-mV	171	6,690	0.187 ± 0.003	0.093 ± 0.004	13 ± 1.5	0.82 ± 0.025	87 ± 1.4
DnaG-mV	157	6,937	0.230 ± 0.005	0.100 ± 0.005	12 ± 1.6	0.88 ± 0.020	88 ± 1.2

a *D*, average diffusion constant of all molecules (μm^2^·s^−1^).

b *D*_1_, diffusion constant of static fraction (μm^2^·s^−1^).

c *F*_1_, percentage of static molecules.

d *D*_2_, diffusion constant of mobile fraction (μm^2^·s^−1^).

e *F*_2_, percentage of mobile molecules.

10.1128/mSphere.00948-20.2 FIG S2 Goodness of GMM fit and best model selection. Probability-probability plots for every type of damage and for every fluorescent protein fusion are displayed. Dark green shows the model that performs better than the other one; in light green is the poorer fitting model. Mean squared error (MSE) and *R*-squared coefficients were used to find that a two-population fit clearly performs better for all cases. On the highlighted area, details of the correlation and step-size histogram are shown along with the fit for both models below the panel. A three-population model was discarded for reasons of overfitting. *R*-squared coefficients were higher than 0.998 for every two-population model. Download FIG S2, TIF file, 1.1 MB. Copyright © 2021 Hernández-Tamayo et al. 2021 Hernández-Tamayo et al. This content is distributed under the terms of the Creative Commons Attribution 4.0 International license.

10.1128/mSphere.00948-20.8 MOVIE S1 Stream acquisition (20-ms intervals) of cells expressing DnaC-mVenus as the sole source of the protein during exponential growth. Images are shown at 50 frames/s (real time). Download Movie S1, AVI file, 7.5 MB. Copyright © 2021 Hernández-Tamayo et al. 2021 Hernández-Tamayo et al. This content is distributed under the terms of the Creative Commons Attribution 4.0 International license.

10.1128/mSphere.00948-20.9 MOVIE S2 Stream acquisition (20-ms intervals) of cells expressing DnaG-mVenus as the sole source of the protein during exponential growth. Images are shown at 50 frames/s (real time). Download Movie S2, AVI file, 9.1 MB. Copyright © 2021 Hernández-Tamayo et al. 2021 Hernández-Tamayo et al. This content is distributed under the terms of the Creative Commons Attribution 4.0 International license.

### DnaC, DnaG, and DnaE respond differentially to stress conditions at the single-molecule level.

Addition of MMC to growing cells did not strongly affect the size of DnaE populations, as was described before (13). If anything, DnaE turned out to be slightly more stably associated with the forks, becoming more static, from 58% ± 2.2% to 63% ± 1.7% (Fig. 5). Conversely, both DnaC and DnaG revealed a decrease in the static population with a concomitant increase in unbound molecules (Fig. 4, second row of panels). For DnaC, the size of the static population observed during exponential growth (81% ± 1.7%) was almost halved to 42% ± 1.6%, while for DnaG, the static population decreased from ∼46% ± 1.9% to 29% ± 1.4% (Fig. 5). Because chromosome segregation and, thus, replication continue after addition of MMC, albeit more slowly (36), likely based on frequent restart processes, it follows that DnaC molecules are more frequently exchanged during replication restart than polymerases. Unexpectedly, blocking of PolC by HPUra leads to an increase of fork-bound primase molecules (Fig. 4, third row), while populations of DnaC remained unchanged after MMC treatment, and DnaE became slightly more dynamic. The static fraction of DnaG almost doubled from ∼46% ± 1.9% to 81% ± 1.6%, while that of DnaC almost halved, from about 81% ± 1.7% to 42% ± 1.5% (Fig. 5 and Table 1). Thus, DnaC and DnaG respond in an opposite manner to blocking of polymerase activity. Interestingly, under this condition, DnaE was partially lost from forks, with the static fraction dropping from ∼58% ± 2.2% to 49% ± 2.3% (Fig. 5).

The induction of the stringent response led to a third pattern of changes, namely, a strong decrease in fork-bound, static molecules, and, thus, a large increase in freely diffusive molecules for all three replication proteins (Fig. 4 and 5). We interpret these findings to mean that (i) stringently blocked forks become highly prone to protein exchange (in agreement with weaker fluorescence observed using epifluorescence microscopy; Fig. 2) and, (ii) in spite of a block in DnaG recruitment, forks do not completely disintegrate but are likely ready to rapidly return to activity when ppGpp levels are lowered. In agreement with *in vitro* findings from Rymer et al. (37), our *in vivo* data show that the induction of the stringent response in bacteria interferes with primer synthesis by preventing binding of DnaG to replication forks. Western blot analyses (Text S1) showed no considerable changes in protein levels between growing and drug-treated cells (Fig. S1F), ruling out effects of expression levels on protein localization.

10.1128/mSphere.00948-20.10 TEXT S1 Molecular and microbiological procedures and Western blotting. Download Text S1, DOCX file, 0.01 MB. Copyright © 2021 Hernández-Tamayo et al. 2021 Hernández-Tamayo et al. This content is distributed under the terms of the Creative Commons Attribution 4.0 International license.

The different behaviors of DnaG and DnaE at the single-molecule level after the different kinds of replication stress induced suggest that they are independently recruited to replication forks. Were they generally recruited in an ensemble manner, we would have expected similar shifts in bound and free populations of molecules under the same stress conditions.

### Nutritional downshift/stringent response increases turnover of helicase, primase, and polymerase at the forks.

**FIG 6:**
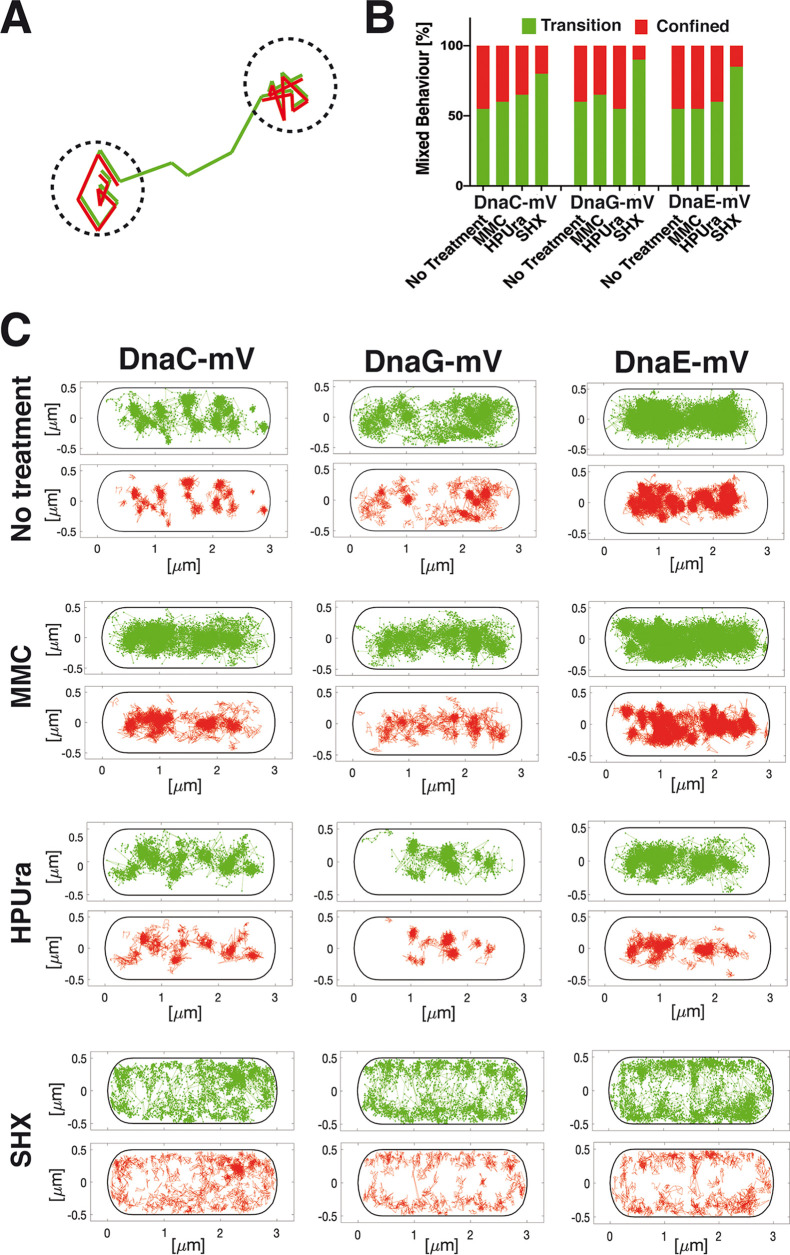
Analyses of tracks for the percentage of transitions under stress conditions. (A) Cartoon showing the mode of analyses of confined movement (red) versus transition events (green). (B) Bar plot showing changes between confined and transitory motion for the three proteins under different stress conditions. (C) Maps showing intracellular location of confined motion (as defined by not leaving a radius of 120 nm for at least 9 steps) and transition events for DnaC-mVenus, DnaG-mVenus, and DnaE-mVenus, projected into a standardized cell 3 by 1 μm in size.

We wished to obtain more information on the exchange rates of the three replication proteins. Therefore, we introduced into SMTracker an analytical tool to quantify the extent of molecules that show transitions between mobile mode and static behavior. Confined motion was defined as molecules staying within a radius of 120 nm (about three times our localization error) for at least 9 steps (Fig. 6A). These states of static motion are indicated in red in Fig. 6. Note that a confined track can be part of a longer track that changes between static and mobile mode or vice versa (Fig. 6A). Such transition tracks are shown in green in Fig. 6. Not shown are tracks of molecules that are entirely mobile/freely diffusive and do not rest for 9 steps in a row (Fig. S3); note that even freely diffusive molecules can stochastically stop for shorter periods of time. Conversely, confined motion occurs when a protein has restricted movement for an extended amount of time, which is due to an interaction with/binding to a much larger subcellular structure. To locate these events, a confinement map tool was developed, using the information given by the dwell time calculation and projecting events into a standardized cell. Figure 6C shows that the confined motion of DnaC, DnaG, and DnaE clusters in the cell center where replication forks are present, as expected. In agreement with the finding that DnaG shows the smallest static fraction, it also showed the smallest extent of confined motion, while DnaE showed the largest (Fig. 6C). Treatment with MMC visually increased the percentage of molecules showing transitions, while HPUra did not appear to strongly affect the ratio between confined tracks and those undergoing transitions (Fig. 6C). To better quantify these findings, we scored tracks undergoing purely diffusive motion, transitions, or purely confined motion. The latter two fractions were set to 100%, and we scored the change between confined and transitory molecules. Figure 6B shows that MMC treatment slightly increased transitions of DnaC at the forks. This was more pronounced during blocking of polymerases by HPUra, in agreement with DnaC becoming more mobile under this condition (Fig. 4 and Fig. S3). Interestingly, transitions greatly increased after the addition of SHX, not only for DnaC but also for DnaG and DnaE (Fig. 6B and C), revealing that during stringent responses, all three proteins revealed highly increased exchange rates at the forks, even though these were blocked for elongation. Of note, B. subtilis cells growing in rich medium at 30°C show overlapping rounds of replication (38); therefore, all cells can be expected to be actively replicating at any time during their cell cycle.

10.1128/mSphere.00948-20.3 FIG S3 Freely diffusive movement of molecules occurs throughout the cell. A confinement map has been developed alongside the information given by the dwell time calculation algorithm of SMTracker. A trajectory is considered to present confinement (red) when it has at least one dwell event. Molecules changing between confinement and mobility are termed “transition” (mixed behavior) and are shown in green. Freely diffusive molecules lacking considerable parts of confinement are shown in blue. Download FIG S3, TIF file, 1.6 MB. Copyright © 2021 Hernández-Tamayo et al. 2021 Hernández-Tamayo et al. This content is distributed under the terms of the Creative Commons Attribution 4.0 International license.

**FIG 7:**
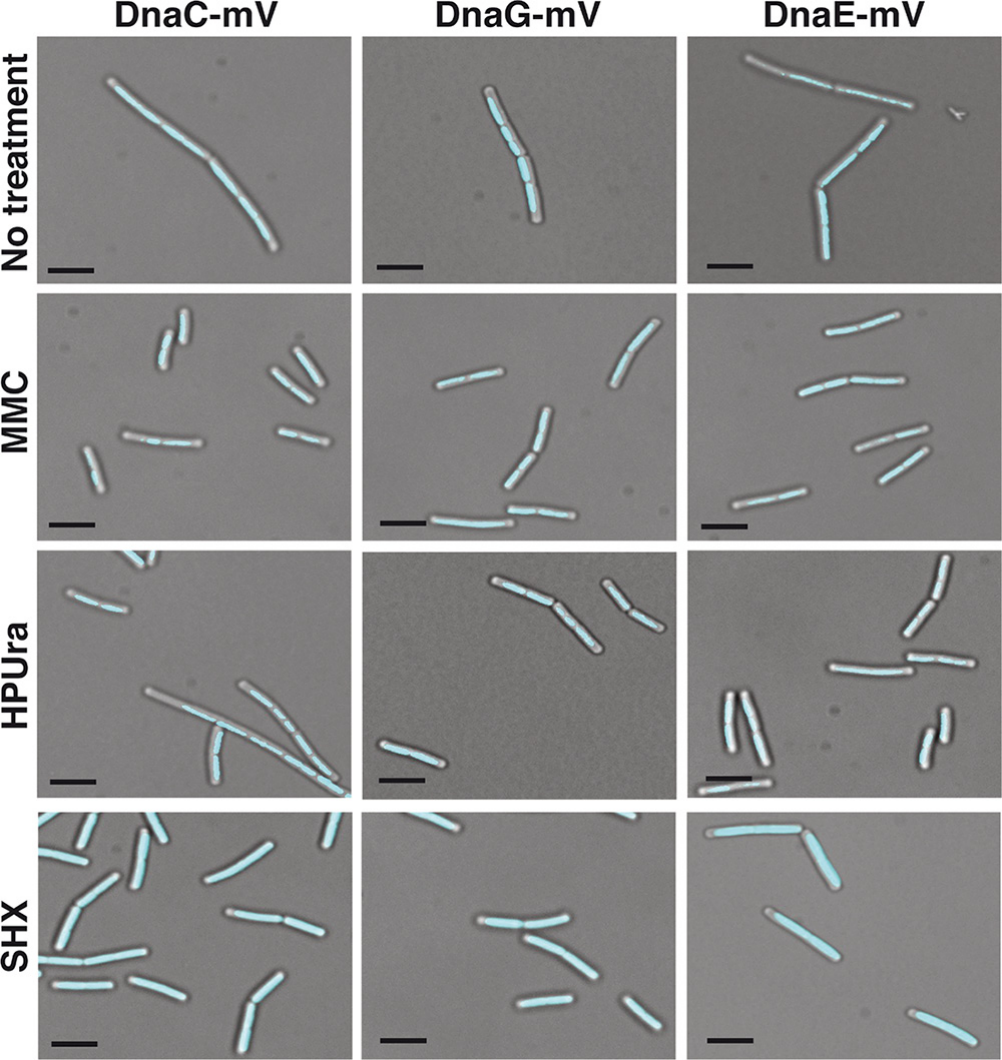
DNA localization patterns. Localization by epifluorescence in representative live B. subtilis cells in untreated cells or in MMC-treated, HPUra-treated, or SHX-treated cells, as stated next to the panels. Black scale bars, 2 μm.

Surprisingly, the pattern of localization of confined and transitory tracks changed markedly under stringent conditions. All three proteins showed localization away from the cell center toward the periphery of cells (Fig. 6). Curious about this finding, we stained cells with 4′,6-diamidino-2-phenylindole (DAPI) to study the subcellular localization of the chromosome(s). Figure 7 shows regular nucleoids during exponential growth and condensed nucleoids following the addition of MMC, as has been reported before (13). Nucleoids were also more condensed after addition of HPUra but were almost completely decondensed during the stringent response (Fig. 7). Previously, it has been shown that 70S ribosomes occupy nucleoid-free zones at the cell poles and at the cell periphery (39), which is lost upon inhibition of transcription, where the nucleoids decondense (40). Our finding that the stringent response also leads to a strong nucleoid decondensation suggests that under this condition, the separation between RNA polymerase and ribosomes is lost. Our data also suggest that the central positioning of replication forks is lost, which agrees with the appearance of more randomly located forks seen in epifluorescence (Fig. 2). Thus, nutritional downshift not only strongly increases exchange of proteins at the replication forks but also affects their subcellular localization.

### Stress conditions differentially affect dwell times of DnaC, DnaG, and DnaE.

**FIG 8:**
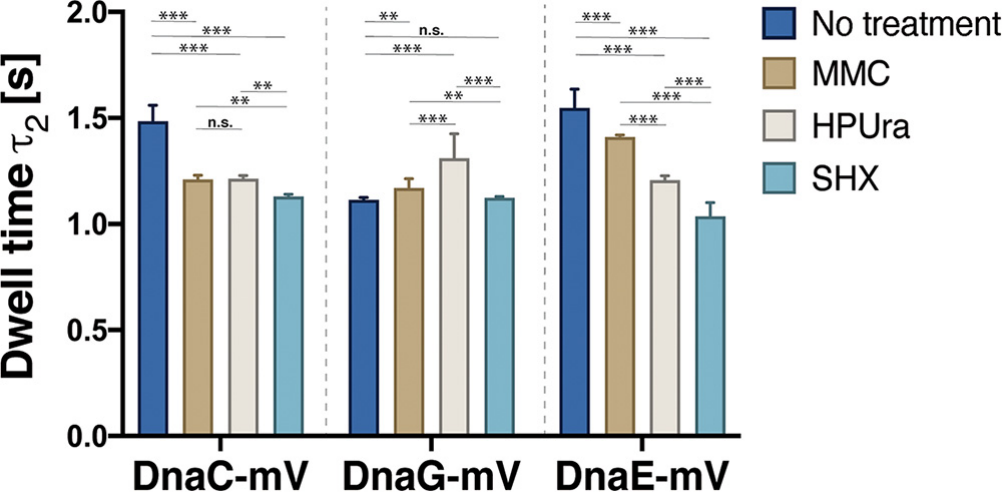
Dwell times. Cumulative distribution of residence times of DnaC-mVenus, DnaG-mVenus, and DnaE-mVenus strains, before and after treatment with MMC, HPUra, or SHX. Dwell times are estimated using an exponential decay model. Histograms show events of resting fitted by a two-component exponential function. Bars represent long dwell times of DNA-bound molecules. Dark blue bars, untreated cells; brown, MMC-treated cells; gray, HPUra-treated cells; and light blue, SHX-treated cells. **, *P* < 0.01; ***, *P* < 0.05; n.s., statistically not significant.

Based on the findings described above, we would have predicted lower dwell times of replication at the forks during nutritional downshift. We analyzed times molecules dwell within a radius of 120 nm and used a two-population fit to analyze decay curves (Fig. S4), which resulted in better fitting than using a single decay function. Fraction τ_1_ shows shorter average dwell times and will mostly consist of mobile molecules that stochastically stay put for a short time. Fraction τ_2_ will likely be composed of molecules residing at the replication machinery. Of note, although average track lengths (around 8 steps) were shorter than average dwell times determined, especially for the second fraction (τ_2_) that shows long dwell times, there were enough tracks longer than average track length to allow for a correct extrapolation of average residence time. Because we used yellow fluorescent protein (YFP)-bleaching-type SMT, our determined numbers are underestimates of actual dwell times *in vivo*. However, for the sake of comparison between different growth conditions, our estimates are useful to observe relative changes in dwell times. Figure 8 shows that, in agreement with our expectations, the dwell time of DnaC decreased during (transient) replication arrest due to MMC or HPUra treatment and further decreased during the stringent response. For DnaG, we observed an increased residence time after addition of HPUra (Fig. 8 and Table S3), corresponding to the increase in the fraction of static molecules observed (Fig. 4). However, under stringent conditions, average residence time remained similar to values during unperturbed growth, which is unexpected. DnaE showed the clearest changes in dwell times, which decreased from MMC to SHX treatment, where residence times were shortest.

10.1128/mSphere.00948-20.4 FIG S4 Survival function of dwell times. For every fluorescent protein fusion displayed, the probability that a molecule will remain inside a circle of radius of 120 nm for time *t* is shown. Dwell times are estimated using an exponential decay model. Blue bars show empirical data, and green and red lines show the 1- and 2-component exponential models fitted to the data. A 1-component decay cannot explain long dwell times; a better result was obtained for a 2-components model. The inset shows the average number of dwell events per length of the track including standard errors. Download FIG S4, TIF file, 2.0 MB. Copyright © 2021 Hernández-Tamayo et al. 2021 Hernández-Tamayo et al. This content is distributed under the terms of the Creative Commons Attribution 4.0 International license.

Keeping in mind the caveat that our determined dwell times are underestimates, we can still observe that DnaC and DnaE show similar residence times during nonperturbed growth. This is surprising, given that helicase DnaB shows much longer dwell times at the single-molecule level than Pol III in E. coli cells (41). We have previously shown that the average bleaching half time of YFP is about 1.3 s in our SMT setup (34) and is expected to be somewhat longer for mVenus, which has been shown to be more resistant to bleaching than its parent, YFP (42). This suggests that while dwell time analyses are certainly convoluted by bleaching, the extent is not overwhelmingly large. Thus, as DnaE exchanges with every 1,000 to 2,000 bp synthesized at the lagging strand, so do DnaC subunits of the hexamer appear to exchange at this DNA strand. We propose that this exchange is based on the exchange of subunits from the hexameric helicase, which appear to exchange within a time frame of a few seconds, supporting the statement of Velten et al. (43), who showed evidence that the DnaC helicase loading mechanism appears to be of the ring-assembly type, proceeding through the recruitment of DnaC monomers and their hexamerization around single-stranded DNA.

## DISCUSSION

All cells need to adjust their decision of when to replicate to the nutritional state of the cell as well as to many other conditions and physiological requirements. Especially for bacteria, it is important to regulate not only initiation of replication but also extension, because a runout of, e.g., nucleotides might be detrimental if replication fork speed was not downregulated. We have sought to shed light on the question of how Bacillus subtilis, a model organism, especially for the large group of Gram-positive bacteria, adjusts replication at the single-molecule level in response to amino acid starvation. Interestingly, the architecture of B. subtilis replication forks is rather similar to that of eukaryotic cells and dissimilar to that of other model bacteria, such as E. coli, in that two replicative polymerases act at the lagging strand (10, 11) rather than one (5, 6), besides other differences.

Recent studies on bacterial DNA replication have supported the idea of a replisome machinery that freely exchanges DNA polymerases (44) and shows strong coupling between helicase and the polymerase(s) (45). Modifying the textbook model of the clamp loader complex acting as a stable hub coordinating the replisome, these observations suggest a role of the helicase as the central organizing hub. We show here that there is a high degree of plasticity in the interaction between the lagging strand polymerase and the replicative helicase upon association of the primase with the replisome. By combining epifluorescence and *in vivo* single-molecule assays, we demonstrate that replicative helicase DnaC, DNA primase DnaG, and lagging-strand polymerase DnaE act differentially in response to transient replication blocks due to DNA damage, to inhibition of the replicative polymerase, or to downshift of serine availability (stringent response).

The addition of HPUra has been shown to block the activity of the replicative DNA polymerase in several bacterial species. Interestingly, we find more static binding of primase DnaG at the forks and an increase in its dwell times, while DnaE becomes slightly more dynamic and helicase DnaC even more so. These findings suggest that blocking of PolC allows for completion of an Okazaki fragment and permits DnaG to reinitiate binding but slows down or blocks its turnover. The exchange of DnaC molecules was found during exponential growth and in an increased manner during all three stress conditions when the progression of the forks was blocked or strongly reduced. We interpret these findings to indicate that the hexameric helicase exchanges its subunits within intervals of a few seconds, replacing them continuously, as is known from exchange of, e.g., rotor parts of the bacterial flagellum (46).

While DnaE became slightly more statically associated with replication forks during MMC-induced DNA repair and less so during blocking of PolC via addition of HPUra, DnaG became highly stabilized during blocking of PolC activity and less statically positioned during DNA repair. Thus, responses in single-molecule dynamics were quantitatively different between DnaG and DnaE during conditions of replication stress. Likewise, dwell times changed in very different manners during stress conditions, indicating that both proteins are recruited separately and independently from each other to initiate new Okazaki fragments. Moreover, although DnaC, DnaG, and DnaE have been shown to form a stable complex *in vitro* (12), changes in single-molecule dynamics of DnaC in response to MMC or HPUra treatment were distinct from those of DnaG and DnaE, showing intriguing plasticity in protein dynamics within this complex and, thus, within replication forks.

The most pronounced changes in single-molecule dynamics were found after nutritional downshift. All three proteins became much less statically associated with forks during the stringent response, concomitant with a decrease in dwell times for DnaC and for DnaE and strongly increased turnover of binding/unbinding events. These findings show that interaction of DnaG with the stringent response second messenger (p)ppGpp (25, 26), which is synthesized via RelA in response to binding of uncharged tRNAs at the ribosome A site (22, 23), strongly reduces binding of DnaG to the lagging strand rather than blocking its activity and stalling DnaG at the forks. Additionally, our findings show that chromosomes decondense during the stringent response, in contrast to condensation during fork block via MMC or HPUra. Interestingly, replication forks persist but were dislocated, no longer occupying central positions within the cell, again contrary to conditions of chemically blocked forks. Most strikingly, stringently arrested forks featured highly increased protein turnover for all three proteins monitored. This will lead to the slowing down of replication elongation, or even halt replication completely, but allow for rapid regaining of extension, possibly without the need for restart.

We have recently shown that chromosome segregation and, thus, DNA replication, which occur concomitantly, are relatively robust against DNA damage induction via MMC or inhibition of DNA gyrase, continuing to follow a general pattern that resembles that of directed diffusion (36). Here, we show that replication forks can be seen to persist during MMC treatment as well as during inhibition of PolC or of DnaG via (p)ppGpp binding. Apparently, while featuring rapid exchange of subunits, the replication machinery appears to be very robust against different kinds of stresses and can be tuned down in speed after nutritional downshift. Clearly, nature has evolved a highly adaptable and overall processive/stable machinery for one of the most central aspects of life, duplication of the genetic information.

## MATERIALS AND METHODS

### Bacterial strains and growth conditions.

The bacterial strains and plasmids used in this study are listed in Table S1 in the supplemental material, and the nucleotides are listed in Table S2. Escherichia coli strain DH5α (Stratagene) was used for the construction and propagation of plasmids. All Bacillus subtilis strains were derived from the wild-type strain BG214. Cells were grown in Luria-Bertani (LB) rich medium at 30°C. When needed, antibiotics were added at the following concentrations (in micrograms per milliliter): ampicillin, 100; chloramphenicol, 5; spectinomycin, 100; kanamycin, 30. When required, media containing 50 ng/ml mitomycin C (MMC), 50 μg/ml 6(p-hydroxyphenylazo)-uracil (HPUra), or 5 mg/ml d,l-serine hydroxamate (SHX) were prepared by adding appropriate volumes of a filter-sterilized solution.

10.1128/mSphere.00948-20.5 TABLE S1 Bacterial strains and plasmids. Download Table S1, DOCX file, 0.02 MB. Copyright © 2021 Hernández-Tamayo et al. 2021 Hernández-Tamayo et al. This content is distributed under the terms of the Creative Commons Attribution 4.0 International license.

10.1128/mSphere.00948-20.6 TABLE S2 Oligonucleotides used in this work. Download Table S2, DOCX file, 0.01 MB. Copyright © 2021 Hernández-Tamayo et al. 2021 Hernández-Tamayo et al. This content is distributed under the terms of the Creative Commons Attribution 4.0 International license.

10.1128/mSphere.00948-20.7 TABLE S3 Average dwell times (in seconds) of DnaC-mVenus, DnaE-mVenus, and DnaG-mVenus. Download Table S3, DOCX file, 0.01 MB. Copyright © 2021 Hernández-Tamayo et al. 2021 Hernández-Tamayo et al. This content is distributed under the terms of the Creative Commons Attribution 4.0 International license.

### Construction of strains.

DnaE, DnaG, and DnaC were visualized as DnaE-mVenus, DnaG-mVenus, and DnaC-mVenus (mV) fusion proteins expressed at the original locus. The last 500 bp coding for each gene were integrated into vector pSG1164-mVenus (47), using ApaI and EcoRI restriction sites, and BG214 cells were transformed with this construct, selecting for Cm resistance (leading to strains listed in Table S1). For colocalization studies, DnaX-CFP was integrated at the *amyE* locus by the use of the plasmid pSG1192, and expression was controlled by xylose addition (48). All fusions contain the linker sequence Ser Gly Gly Ser Gly Gly Ser Gly Gly. To investigate colocalization of DnaE, DnaG, and DnaC, the resulting strains PG3307, PG3322, and PG3323 (Table S1) were transformed with chromosomal DNA of strains leading to the expression of DnaX-CFP in parallel to DnaE-mV, DnaG-mV, or DnaC-mV.

### Flow cytometry.

Exponentially growing cells (optical density at 600 nm [OD_600_] of 0.5 to 0.7) were stained using Vybrant DyeCycle orange stain (VDCO) (V35005; Invitrogen) at a final concentration of 10 μM at 37°C for 30 min according to the sample treatment. Cells were diluted 10-fold in phosphate-buffered saline before DNA content measurement by flow cytometry (BD LSR Fortessa; Becton, Dickinson GmbH, Heidelberg, Germany). About 20,000 cells were analyzed for each data set. The peaks corresponding to cells with one, two, or more chromosome contents were identified by comparison to the standard (BG214 cells). Technical triplicates were analyzed per strain.

### Growth and survival studies.

A single colony of B. subtilis cells was inoculated in 2 ml LB and grown overnight (ON) at 30°C; the culture was then diluted to an OD_600_ of 0.05 and grown to an OD_600_ of 0.8 in LB broth as described previously (49). Cultures were treated with 50 ng/ml MMC, 50 μg/ml HPUra, or 5 mg/ml SHX. Incubation was done for 60 min at 30°C for MMC and HPUra, and 15 min for SHX. Parallel cultures were performed in the absence of drugs. For the survival assay, cells were grown to reach exponential phase (OD_600_ of 0.4) at 30°C and treated with or without drugs as stated above, and appropriate dilutions were plated on LB plates. Plates were grown ON (16 to 18 h) at the indicated temperature.

### Fluorescence microscopy.

For fluorescence microscopy, B. subtilis cells were grown in LB at 30°C under shaking conditions until exponential growth. Conventional light microscopy was performed using a Zeiss Observer Z1 (Carl Zeiss) with an oil immersion objective (×100 magnification, 1.45 numerical aperture; alpha Plan-FLUAR; Carl Zeiss) and a charge-coupled device (CCD) camera (Cool SNAP EZ; Photometrics). Data were processed using MetaMorph 7.5.5.0 software (Molecular Devices, Sunnyvale, CA, USA). When required, cells were incubated with 50 ng/ml MMC, 50 μg/ml HPUra, or 5 mg/ml SHX. Incubation was for 60 min at 30°C before microscopy.

### Single-molecule microscopy and tracking.

Cells were spotted on coverslips (25 mm; Menzel) and covered using 1% agarose pads prepared before with fresh S7_50_ minimal medium by sandwiching the agarose between two smaller coverslips (12 mm; Marienfeld). All coverslips were cleaned before use by sonication in Hellmanex II solution (1%, vol/vol) for 15 min, followed by rinsing in distilled water and a second round of sonication in double-distilled water. In contrast to the wide-field illumination used in conventional epifluorescence microscopy, the excitation laser beam used in our setup is directed to underfill the back aperture of the lens objective, generating a concentrated parallel illumination profile at the level of the sample, leading to a strong excitation followed by rapid bleaching of the fluorophores. When only a few unbleached molecules are present, their movement can be tracked. In addition, freshly synthesized and folded fluorophores become visible when the sample is excited again. When an observed molecule is bleached in a single step during the imaging, it is assumed to be a single molecule (5, 50). Image acquisition was done continuously during laser excitation with the electron-multiplying CCD (EMCCD) camera iXon Ultra (Andor Technology, Belfast, UK). A total of 2,500 frames were taken per movie, with an exposure time of 20 ms (23 fps). The microscope used in the process was an Olympus IX71 with a 100× objective (UAPON 100×OTIRF; numerical aperture, 1.49; oil immersion). A 514-nm laser diode was used as the excitation source, and the band corresponding to the fluorophore was filtered out. Of note, cells continued to grow after imaging, showing that there is little to no photodamage during imaging, while cells stop growing when exposed to blue light (below 480 nm). Acquired streams were loaded into Fiji ImageJ (51). Automated tracking of single molecules was done using the ImageJ plugin MtrackJ or u-track 2.2.0 (35).

### Diffusion analysis of single-molecule tracks.

Tracking analysis was done with u-track-2.2.0, which was specifically written for Matlab (MathWorks, Natick, MA, USA). Only trajectories consisting of a minimum of 5 frames were considered tracks and included for further analysis. A widely accepted method to analyze the diffusive behavior of molecules is by using the curve of mean squared displacement (MSD) versus time lag (52, 53). This provides an estimate of the diffusion coefficient as well as of the kind of motion, e.g., diffusive, subdiffusive, or directed. However, the method requires that within a complete trajectory there be only one type of homogeneous motion and that the trajectory is preferably of infinite length. To distinguish immobile and mobile molecules from each other, we compare the frame-to-frame displacement of all molecules in *x* and *y* directions. We used a Gaussian mixture model (GMM) to fit the probability density distribution function of all frame-to-frame displacements, determine the standard deviations σ_1_ and σ_2_, and the percentages *F*_1_ and *F*_2_ of the slow and the fast subfractions of molecules, respectively. Finally, the diffusion constants were calculated according to $D_{i}=\frac{\sigma^{2}}{2△t},\left(i=1,2\right)$, where *Δt* is the time interval between subsequent imaging frames.

Generation of heat maps, analyses of molecule dwell times, and visualization of slow and fast tracks in a standardized cell are based on a custom-written Matlab script (SMTracker) that is available on request (34). SMTracker can use particle-tracking tools u-track (35) and TrackMate (54) and computes the *x* and *y* coordinates of molecular trajectories relative to the geometry of each cell, as obtained by the cell segmentation tool MicrobeTracker (55) or Oufti (56).

### Dwell times.

Dwell time is defined as the average duration that a particle stays inside a certain region. Observing the trajectories in this manner could give insights, for example, on how long the replication proteins are bound at the replication fork. For that matter, dwell time calculations need as parameters the (circular) region and the minimum number of steps that a molecule should remain inside the region (1 step equals 1 time interval). The procedure operates in such a way that searches for the longest dwell events of the protein in each trajectory. For *T* = (*C*_1_, … *C_n_*), a trajectory is defined as a set of nodes, *C_i_* = (*x_i_*, *y_i_*), where *x_i_* and *y_i_* are the nodes’ coordinates in a Cartesian axis, and the circle *C*(*C_k_*, *R*) is chosen, with *R* being the radius that contains the maximum number of consecutive points of the trajectory. The amount of time the molecule stays then is counted, and the same track *T* excluding that segment of trajectory, *T*/(*C_k_*, … *C_k+p_*), is again searched for more dwell events. The procedure finishes when no more dwell events can be found. In our procedure, one gap (point absent for one frame) or one point outside the circle that goes and comes back is also considered to have remained inside the circle. The number of dwell events and their frequency is plotted in a pdf histogram, and the data are fitted to a single or, if appropriate, multiexponential decay to distinguish up to two different populations of dwell time events.

### Statistical data analysis.

The goodness of fits of the Gaussian mixture models was assessed using probability-probability plots (pp-plots). Errors on the fitted parameters are given as 95% confidence intervals, which were derived from the Jacobian matrix of the nonlinear optimization process using the MATLAB function *nlparci.* To compare fraction sizes and diffusion constants under different conditions and between different proteins, statistical hypothesis testing was performed using *Z* tests. Differences in dwell time and step size distributions were tested using a Kolmogorov-Smirnov 2-sample test. To assess the most likely number of populations for each fit, we applied the Bayesian information criterion (BIC), as detailed in reference 34. *P* values lower than or equal to 0.05 (**) and 0.001 (***) were considered significant, while n.s. means statistically not significant. Statistical hypothesis testing and plotting were performed using SMTracker (34, 57) and MATLAB custom scripts.
