# Supplementary figures and images for: Single-Molecule Dynamics at a Bacterial Replication Fork after Nutritional Downshift or Chemically Induced Block in Replication
Source: mSphere. 2021 Jan 27;6(1):e00948-20. doi: 10.1128/mSphere.00948-20 (PMC7885319; doi:10.1128/mSphere.00948-20)

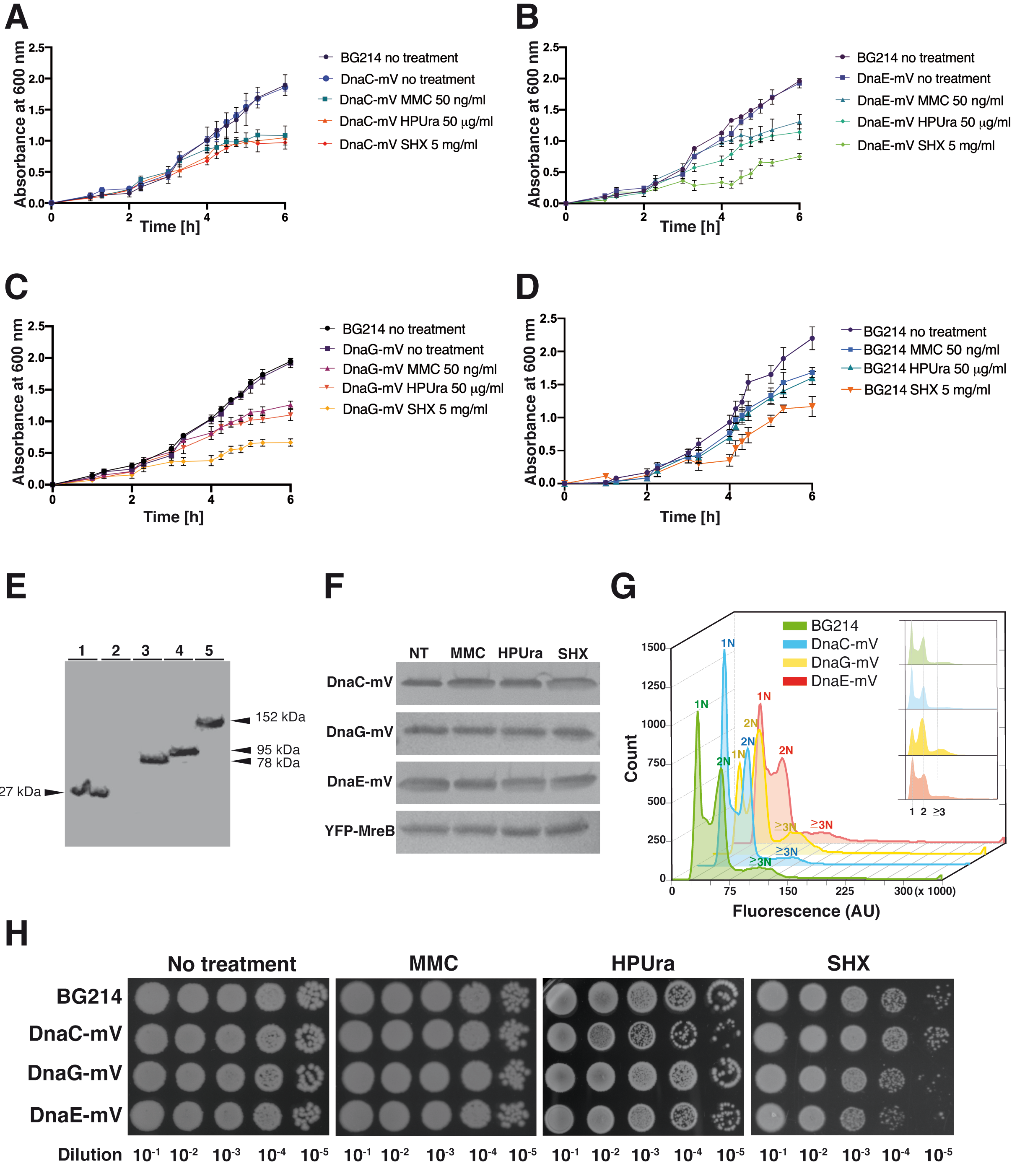

Supplement: FIG S1 [file mSphere.00948-20-sf001.tif]

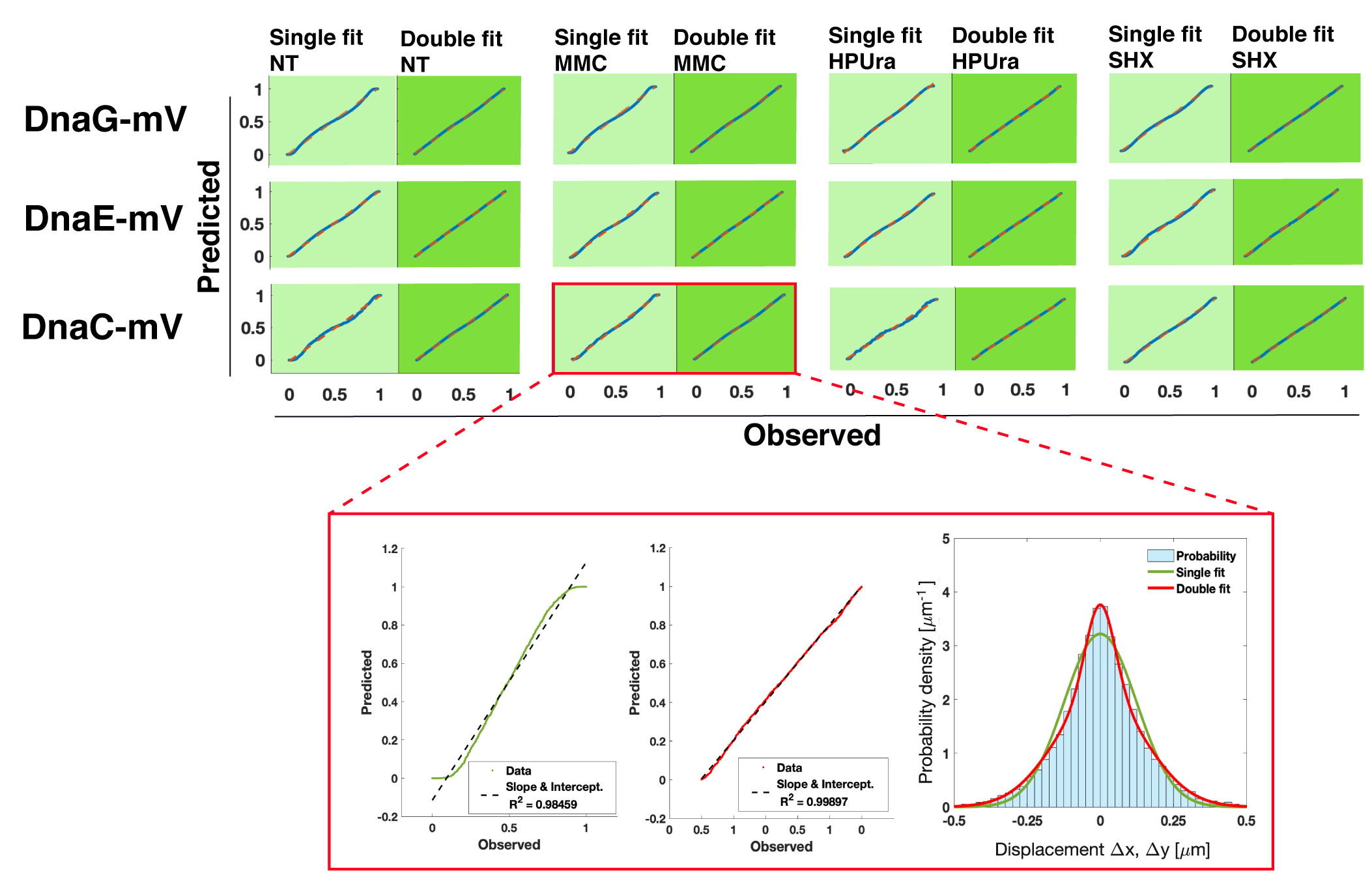

Supplement: FIG S2 [file mSphere.00948-20-sf002.tif]

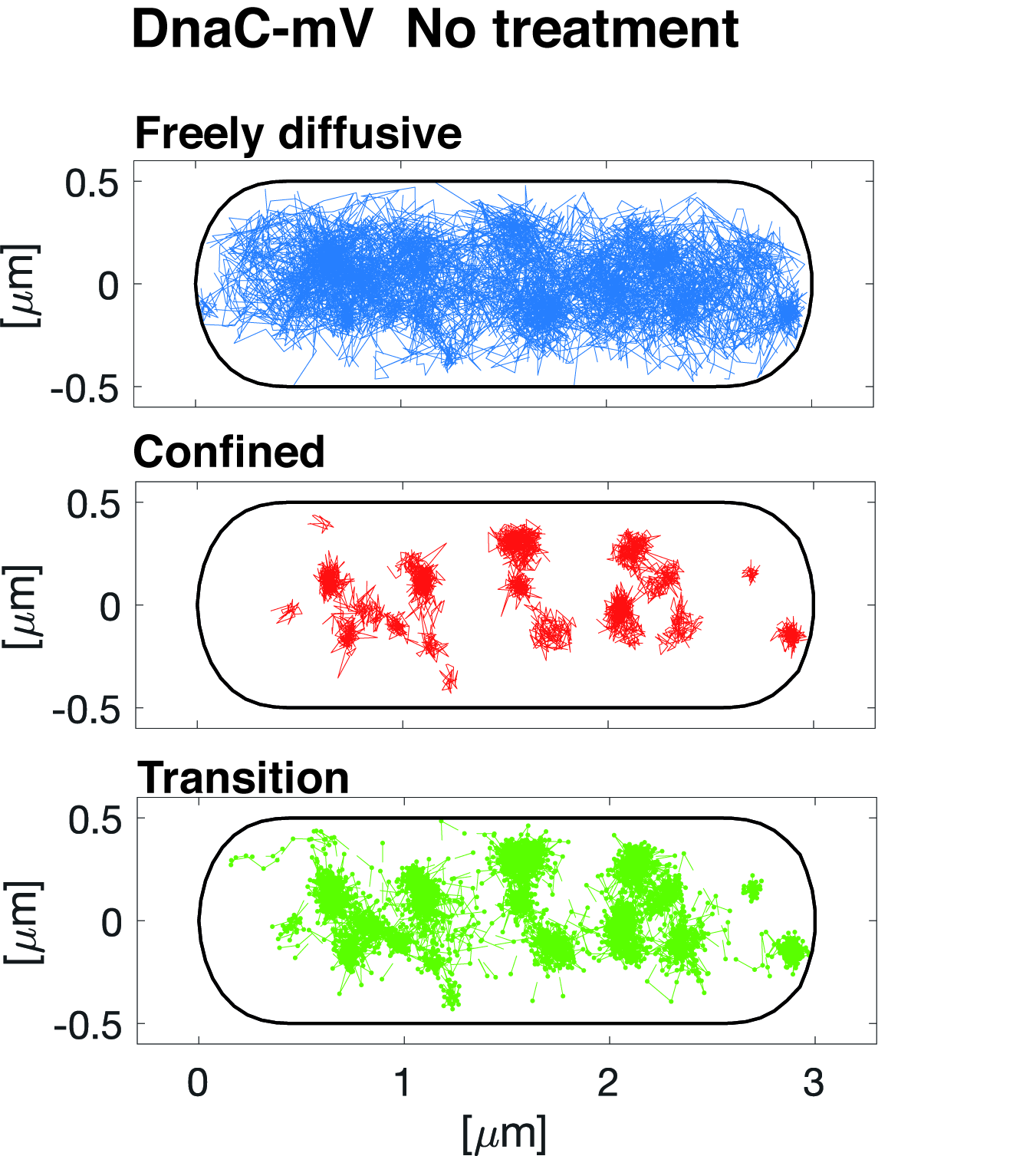

Supplement: FIG S3 [file mSphere.00948-20-sf003.tif]

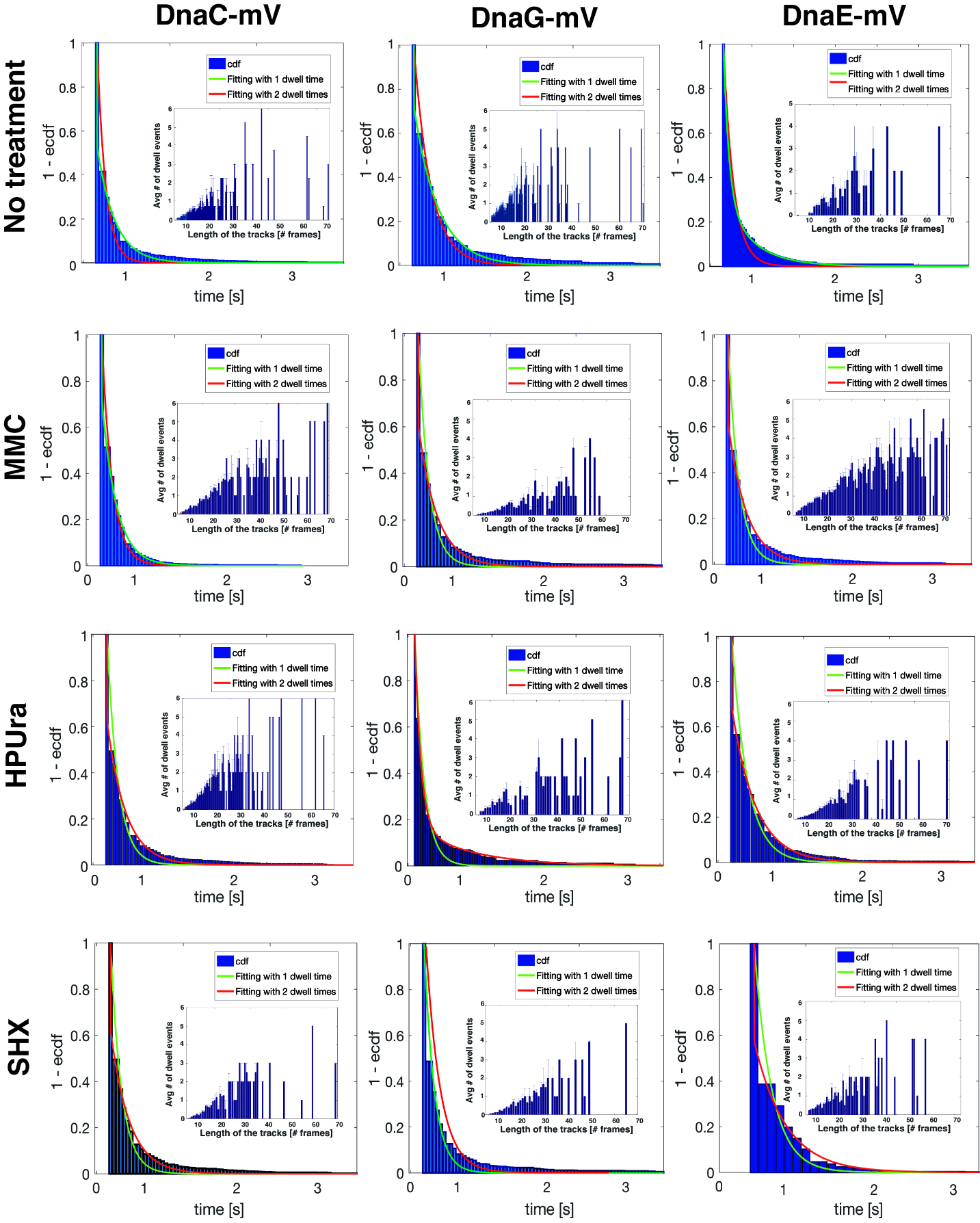

Supplement: FIG S4 [file mSphere.00948-20-sf004.tif]
