# Supplementary material for: Single-Molecule Dynamics at a Bacterial Replication Fork after Nutritional Downshift or Chemically Induced Block in Replication
Source: mSphere. 2021 Jan 27;6(1):e00948-20. doi: 10.1128/mSphere.00948-20 (PMC7885319; doi:10.1128/mSphere.00948-20)
Supplement: TEXT S1 [file mSphere.00948-20-s0001.docx]

**Supplemental methods**

Molecular and microbiological procedures.

Basic DNA manipulations and molecular techniques were done using established procedures (1). To generate specific DNA fragments suitable for cloning, all PCR amplifications were carried out using *Taq* polymerase High Fidelity (New England Biolabs). Amplification protocols consisted of 30 cycles of 1 minute at 94°C, 1 minute at variable temperature (depending on the primer combination, and 1 to 3 minutes at 68°C. The DNAs were resuspended in Tris-EDTA buffer and digested with the appropriate restriction enzyme(s) to generate the required ends of the fragments. The DNA fragments were purified before cloning by isolating them from agarose gels. Primer combinations (Table S2) employed to generate fragments for cloning and electrophoretic mobility shift analyses were generated by annealing custom-made oligonucleotides, purified using a GeneJET extraction kit (QIAGEN). For ligations, T4 polynucleotide ligase (New England Biolabs) was used. Plasmid transformation of *E. coli* was done using CaCl_2_-competent cells. All plasmid constructions were verified by restriction analysis and PCR and in all of the cases, by DNA sequencing.

Western blotting.

*B*. *subtilis* cultures (1 ml) were harvested by centrifugation. The pellet was resuspended in lysis buffer (20 mM Tris-HCl [pH 7.0], 10 mM EDTA, 1 mg ml^-1^ lysozyme, 10 g ml^-1^ DNase I, 100 g ml^-1^ RNase I, 1 tablet of Mini EDTA-free, EASY pack (Roche, protease inhibitor cocktail), and incubated for 30 min at 37°C. Proteins were separated by running 12% sodium dodecyl sulfate-polyacrylamide gel electrophoresis (SDS-PAGE) and were transferred onto nitrocellulose membrane followed by blocking with 5% milk in PBST (80 mM Na_2_HPO_4_, 20 mM NaH_2_PO_4_, 100 mM NaCl, 0.2% (v/v) Tween-20). Proteins were probed using a 1:500 dilution (rabbit-α-GFP) and secondary antibody was added (goat-α-rabbit-antibody in 1:10000 dilution) after a series of washing steps with PBST. Solution A (100 mM Tris pH 8.5, 2.5 mM Luminol, and 0.4 mM Coumaric acid) and Solution B (100 mM Tris pH 8.5, 0.02% (v/v) H_2_O_2_) were prepared and mixed followed by incubation for 2 min for chemiluminescence detection with ChemiDocTM MP System (BIO-RAD).

**Reference**

1. Chong L. 2001. Molecular cloning - A laboratory manual, 3rd edition. Science 292:446-446.
