# Supplementary material for: Single-Molecule Dynamics at a Bacterial Replication Fork after Nutritional Downshift or Chemically Induced Block in Replication
Source: mSphere. 2021 Jan 27;6(1):e00948-20. doi: 10.1128/mSphere.00948-20 (PMC7885319; doi:10.1128/mSphere.00948-20)
Supplement: TABLE S1 [file mSphere.00948-20-st001.docx]

**TABLE S1** Bacterial strains and plasmids.

| Strain or plasmid | Relevant features | | Reference or source |  |
| --- | --- | --- | --- | --- |
| *B. subtilis* |  |  | | |
| BG214 | Wild type |  | | |
| PG3300 | amyE::psg1192 *dnaX*-cfp^specR^ | (1) | | |
| PG3320 | *dnaC*-mVenus^cmR^ | This study | | |
| PG3302 | *dnaE*-mVenus^cmR^ | (1) | | |
| PG3321 | *dnaG*-mVenus^cmR^ | This study | | |
| PG3322 | *dnaX-*cfp^specR^ *dnaC*-mVenus^cmR^ | This study | | |
| PG3307 | *dnaX-*cfp^specR^ *dnaE*-mVenus^cmR^ | (1) | | |
| PG3323 | *dnaX-*cfp^specR^ *dnaG*-mVenus^cmR^ | This study | | |
| *E. coli* |  |  | | |
| DH5α | *sup*E44 Δ*lac*U169 φ80d*lac*ZΔM15 *hsd*R171 *recA*1 *endA*1 *gyrA*96 *thi-*1 *relA1* | New England Biolabs  (NEB) | | |
| PG3324 | DH5α pSG1164::*dnaC*-mVenus^cmR^ | This study | | |
| PG3325 | DH5α pSG1164::*dnaG*-mVenus^cmR^ | This study | | |
| PG3319 | DH5α pSG1192::*dnaX*-cfp^specR^ | (1) | | |
| PG3315 | DH5α pSG1164::*dnaE*-mVenus^cmR^ | (1) | | |

1. Hernandez-Tamayo R, Oviedo-Bocanegra LM, Fritz G, Graumann PL. 2019. Symmetric activity of DNA polymerases at and recruitment of exonuclease ExoR and of PolA to the Bacillus subtilis replication forks. Nucleic Acids Res 47:8521-8536
