# Supplementary material for: Single-Molecule Dynamics at a Bacterial Replication Fork after Nutritional Downshift or Chemically Induced Block in Replication
Source: mSphere. 2021 Jan 27;6(1):e00948-20. doi: 10.1128/mSphere.00948-20 (PMC7885319; doi:10.1128/mSphere.00948-20)
Supplement: TABLE S2 [file mSphere.00948-20-st002.docx]

**TABLE S2** Oligonucleotides used in this work.

| Name | Sequence^a,b^ | Construct |
| --- | --- | --- |
| 22502 | *C*GAATTCATGTCTTTTGTTCACCTGCA | PG3315 |
| 22503 | *A*GGGCCCTTACCACTGTTTTAAAACGA | PG3315 |
| 22604 | *AC*GAATTCGATTTACATCGATGATACAC | PG3324 |
| 22605 | *A*GGGCCCTGCGCCGGGCGGAACGCCTG | PG3324 |
| 22606 | *C*GAATTCGCTGACAATAGCGGTGAAAC | PG3325 |
| 22607 | *A*GGGCCCTTTTAAAGATCGGTTCAATG | PG3325 |
|  |  |  |

^a^ Non-encoded bases introduced as clamps are shown in italics. Restriction sites are underlined; the oligonucleotides carry either *Eco*RI (GAATTC) or *Apa*I (GGGCCC) sites.

^b^ The location is indicated by the first 5’ nucleotide and the replicon where the sequence is located. Accession numbers are *dnaC* (CP053102 REGION: 4257943…4259307), *dnaE* (CP053102 REGION: 3104280…3107627) and *dnaG* (CP053102 REGION: 2714536…2716347) of *B. subtilis.*
