# Supplementary material for: Single-Molecule Dynamics at a Bacterial Replication Fork after Nutritional Downshift or Chemically Induced Block in Replication
Source: mSphere. 2021 Jan 27;6(1):e00948-20. doi: 10.1128/mSphere.00948-20 (PMC7885319; doi:10.1128/mSphere.00948-20)
Supplement: TABLE S3 [file mSphere.00948-20-st003.docx]

**TABLE S3.** Average dwell times (in seconds).

|  | **No treated** | **MMC** | **HPUra** | **SHX** |
| --- | --- | --- | --- | --- |
| **DnaE-mVenus** | | | | |
| **τ_1_** | 0.086±0.05 | 0.088±0.07 | 0.086±0.04 | 0.064±0.03 |
| **τ_2_** | 1.52±0.2 | 1.4±0.13 | 1.25±0.14 | 1.01±0.18 |
| **Fraction τ_1_ (%)** | 23±0.64 | 26±0.83 | 6±0.94 | 3±0.85 |
| **Fraction τ_2_ (%)** | 77±0.54 | 74±0.67 | 94±0.73 | 97±0.63 |
| **DnaC-mVenus** | | | | |
| **τ_1_** | 0.11±0.05 | 0.081±0.09 | 0.082±0.05 | 0.071±0.05 |
| **τ_2_** | 1.45±0.2 | 1.19±0.18 | 1.20±0.10 | 1.13±0.10 |
| **Fraction τ_1_ (%)** | 28±0.45 | 19±0.46 | 15±0.53 | 11±0.51 |
| **Fraction τ_2_ (%)** | 72±0.74 | 81±0.63 | 85±0.72 | 89±0.64 |
| **DnaG-mVenus** | | | | |
| **τ_1_** | 0.071±0.04 | 0.067±0.04 | 0.011±0.05 | 0.075±0.05 |
| **τ_2_** | 1.11±0.12 | 1.15±0.18 | 1.29±0.2 | 1.11±0.15 |
| **Fraction τ_1_ (%)** | 29±0.42 | 19±0.40 | 30±0.38 | 32±0.65 |
| **Fraction τ_2_ (%)** | 71±0.62 | 81±0.55 | 70±0.64 | 68±0.56 |
